# A stability indicating method development and validation of a rapid and sensitive RP-HPLC method for Nintedanib and its application in quantification of nanostructured lipid carriers

**DOI:** 10.12688/f1000research.138786.1

**Published:** 2023-10-20

**Authors:** Varalakshmi Velagacherla, Yogendra Nayak, K Vijaya Bhaskar, Usha Yogendra Nayak

**Affiliations:** 1 Department of Pharmaceutics, Manipal College of Pharmaceutical Sciences, Manipal Academy of Higher Education, Manipal, Karnataka, 576104, India; 2 Department of Pharmacology, Manipal College of Pharmaceutical Sciences, Manipal Academy of Higher Education, Manipal, Karnataka, 576104, India; 3 Department of Pharmaceutical Chemistry, Manipal College of Pharmaceutical Science, Manipal Academy of Higher Education, Manipal, Karnataka, 576104, India

**Keywords:** Nintedanib, RP-HPLC, Validation, Stress degradation, Nanostructured lipid carriers, Entrapment efficiency

## Abstract

**Background:** Nintedanib (NTB) is a multiple tyrosine kinase inhibitor, been investigated for many disease conditions like idiopathic pulmonary fibrosis (IPF), systemic sclerosis interstitial lung disease (SSc-ILD) and non-small cell lung cancer (NSCLC). NTB is available as oral capsule formulation, but its ability to detect degradants formed through oxidative, photolytic and hydrolytic processes makes it difficult to quantify. In the current work, a novel reversed-phase high-performance liquid chromatography (RP-HPLC) method was developed and validated.

**Methods:** The developed method is simple, precise, reproducible, stable and accurate. The inherent stability of NTB was evaluated using the proposed analytical method approach and force degradation studies were carried out. NTB was separated chromatographically on the Shimadzu C _18_ column as stationary phase (250 ×4.6 mm, 5 µm) using an isocratic elution method with 0.1% v/v triethyl amine (TEA) in HPLC grade water and acetonitrile (ACN) in the ratio 35:65% v/v. The mobile phase was pumped at a constant flow rate of 1.0 ml/min, and the eluent was detected at 390 nm wavelength.

**Results:** NTB was eluted at 6.77±0.00 min of retention time (t _R_) with a correlation coefficient of 0.999, the developed method was linear in the concentration range of 0.5 µg/ml to 4.5 µg/ml. The recovery rate was found to be in the range of 99.391±0.468% for 1.5 µg/ml concentration. Six replicate standards were determined to have an % RSD of 0.04.

**Conclusion:** The formulation excipients didn’t interfere with the determination of NTB, demonstrating the specificity of the developed method. The proposed approach of the analytical method developed can be used to quantify the amount of NTB present in bulk drugs and pharmaceutical formulations.

## Introduction

Nintedanib (NTB) is an oral multiple tyrosine kinase inhibitor that competitively inhibits vascular endothelial growth factor, fibroblasts growth factor receptor, and platelet-derived growth factor receptor due to the nature of its ATP-binding pocket property. ^1^ NTB is used to treat a variety of illnesses, including Idiopathic pulmonary fibrosis (IPF), Systemic sclerosis-associated lung disease, non-small cell lung cancer (NSCLC), several Interstitial Lung Diseases, and COVID-19 linked with IPF. NTB works by preventing the cascaded autophosphorylation of tyrosine kinase receptors. ^1^ NTB has an anti-angiogenic action, making it a potential drug for cancer treatment. It is selectively used in conjunction with docetaxel to treat NSCLC. ^2^ NTB is given at a dose of 120–150 mg and the dosage are once or twice a day. NTB is classified as a class II drug under the biopharmaceutical classification system (BCS). Due to first-pass metabolism by enzymes including CYP3A4 and P-gp inhibition, NTB has an extremely low oral bioavailability of 4.7%. NTB reaches its peak plasma concentration in 2-4 hours with 97.8% protein binding. NTB has a half-life of approximately 9.5 hours when it is given orally. ^3^ With a dose of 150 mg twice daily NTB, uncommon colitis is also seen in certain patients. ^4^

**Figure 1.**
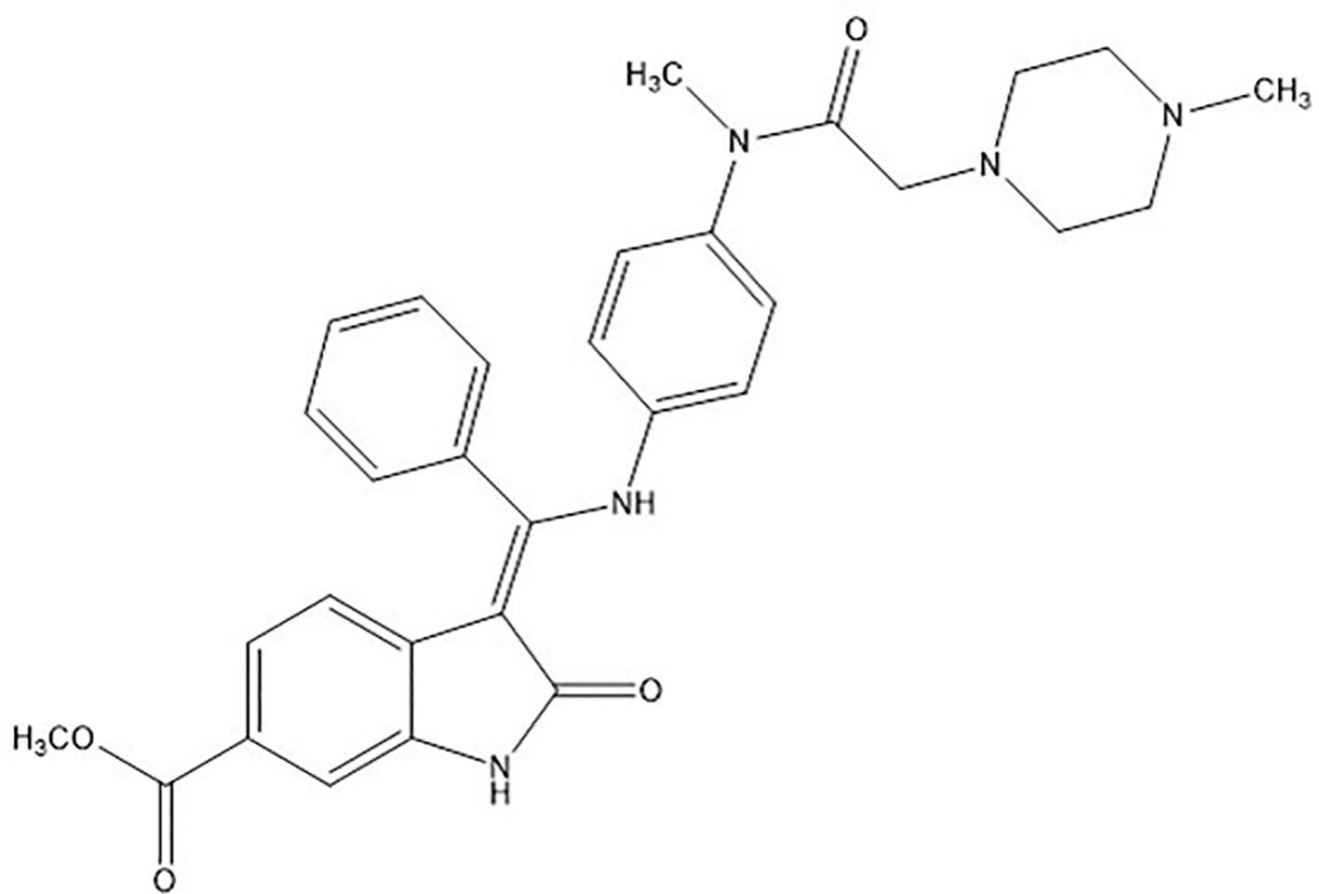
Chemical structure of Nintedanib (NTB).

NTB is having very low aqueous solubility of 0.0309 mg/ml with pKa of 7.23 The chemical structure of NTB is represented in Figure 1. A patent is available on NTB quantification by using high sensitivity analytical method through high-performance liquid chromatography (HPLC) mass spectrometry where they have used formic acid, methanol and ammonium salt as mobile phase and silica gel as stationary phase. ^5^ Pasquini et al have reported a quality by design (QbD) based LC-MS method for estimating NTB and its impurities in soft gelatin capsules. ^6^ Several investigators have reported various methods like RP-UPLC, ^7^ LC-MS, ^8^ RP-HPLC in formulation, and determination of NTB and its stress degradation, ^9^ and which help in estimating the NTB. Some of the methods also reported degradation pathways and the degradation nature of NTB in various solvents with time. As per the existing literature search, none of the methods have reported the usage of the lower organic phase, salt-free analysis, less retention time and less flow rate. These are the parameters that we have focused upon to produce a cost-effective and less time-consuming method. ^9^^,^^10^ The method is precise, rapid, simple and accurate to estimate NTB as per ICH Q2 (R1) guidelines. ^8^ The developed method is used to quantify NTB in Nanostructured lipid carriers (NLCs) loaded with NTB. NLCs are the modified form of solid lipid nanoparticles. NLCs are the combination of liquid and solid lipids in a particular ratio and are known as hybrid nanocarriers. NLCs possess advantages like less particle size, high drug loading, improved solubility, permeation, increased stability and bioavailability. They also have the property of bypassing first-pass metabolism as the absorption takes through the lymphatic system but not from the stomach. ^11^ Some of the lipids like oleic acid and linolic acid which are used in the formulation of NLCs have properties of inhibiting P-gp protein. ^12^ Thus, this current study deals with the development of the analytical method and its validation along with degradation studies and the application of the developed analytical method for estimating the NTB in NLCs based nano-formulations.

## Methods

### Instrumentation and reagents

NTB ≥98% (HPLC) was procured from Sigma-Aldrich, Triethylamine (TEA), orthophosphoric acid (OPA), acetonitrile (ACN) and sodium hydroxide (NaOH) were procured from Merck Ltd. (Mumbai, India). Hydrochloric acid (HCl) is from Loba Chemie (Mumbai, India) and hydrogen peroxide (H _2_O _2_) 30% v/v was procured from Himedia Labs (Mumbai, India). Methanol was got from Finar Ltd. (Ahmedabad, India). HPLC grade water (milli Q water) was procured from the Millipore Direct-Q3 system in the lab (Millipore Pvt. Ltd., Bangalore, India).

Standards and chemicals were weighed by using a sensitive balance (Sartorius AG, Germany). Mobile phases were filtered by using membrane filters 0.22 μm. Mobile phase pH was checked by pH meter (Eutech Instruments pH 510) and sonicated by ultrasonic bath sonicator (GT Sonic, Servewell Instruments). The analytical method was developed in HPLC, Shimadzu LC-2010 CHT (Shimadzu Corporation, Kyoto, Japan) make with quaternary pumps, autosampler, degasser, column oven and dual-wavelength prominence photodiode array (PDA) and ultraviolet (UV) detectors.

### Method development

For the development of an analytical method, different columns like Luna, Phenomenex, Chromasol and Shimadzu C _18_ were used. Effect of various mobile phases like pH 4.5 acetate buffer, pH 6.8 phosphate buffer, 0.1% Trifluoroacetic acid (TFA) and 0.1% TEA were used along with organic solvent ACN. The effect of mobile phase flow rate and temperature of the column was also considered for the method development. The method was optimized by checking all parameters like tailing factor, replicates of standard solution, percentage relative standard deviation (%RSD), theoretical plate count, amount of organic solvent ratio to the aqueous mobile phase, run time and asymmetry of peaks.

### Chromatographic conditions

The isocratic method was used for the analytical method development. Shimadzu C _18_ column was stationary phase and the mobile phase was phosphate buffer (0.1% TEA and the pH was adjusted to 3 with OPA) and ACN with a wavelength of 390 nm at the operating temperature of 25°C. The flow rate of the mobile phase was 1ml/min and injection volume was 20 μl. Each sample run time was 10 min. The optimized chromatographic conditions for the analytical method are given in Table 1.

**Table 1.** Optimized chromatographic conditions for the analytical estimation of NTB.

Specifications	Analytical estimation		
Column	Shimadzu C _18_		
Mobile phase	(0.1% v/v TEA, pH 3.0)		
Isocratic composition	**Time (min)**	**ACN**	**Buffer**
	10	35	65
Flow rate	1 ml/min		
Wavelength	390 nm		
Column temperature	25°C		
Injection volume	20 μl		

ACN: Acetonitrile, TEA: Triethylamine.

### Preparation of working standard

Accurately weighed 5 mg of NTB was added to a 10 ml volumetric flask and dissolved in 80% of methanol in Milli Q water. Further dilutions were made to get 1 μg/ml concentration and from this stock solution, the remaining concentrations were prepared.

### Preparation of formulation samples

10 mg NTB containing nanostructured lipid carriers were centrifuged and the supernatant was decanted where the pellet was dissolved in 1 ml of methanol and necessary dilutions were made with diluent (methanol).

### Method optimization

**Selection of wavelength**

Wavelength was determined by preparing a primary stock solution of NTB (2 mg/ml) in diluent and further dilutions were made to acquire a working standard NTB solution of 2 μg/ml. A UV–Visible spectrophotometer (UV-1601PC, Shimadzu, Japan) was used to scan the NTB standard solution in between the wavelength range of 190-800 nm which helps in the determination of absorption maxima (λ _max_) where water was used as blank.

### Method validation

All the parameters of the developed analytical method were validated according to ICH Q2(R1) regulatory guidelines.

### System suitability

Six samples of 1.5 μg/ml concentration were injected and analyzed by the developed method. After analysis, theoretical plates, tailing factor, retention time and % RSD were calculated from peaks. ^13^

### Specificity

Blank and sample solutions were injected separately, and the peaks were seen to determine the specificity. ^14^

### Linearity

To estimate the range and linearity of the analytical method, various concentrations of NTB solutions (0.5 to 4.5 μg/ml) were prepared. Each concentration was analyzed three times (n=3) under the same chromatographic conditions. The calibration curve linearity was evaluated by linear regression analysis. ^15^

### Precision

Precision of the analytical method was determined by performing analysis of six samples on two different days intraday and inter-day with three concentrations (0.5, 2 and 4 μg/ml) and the range of concentrations were taken from the linearity curve which was categorized into lower quality control (LQC), middle quality control (MQC) and higher quality control (HQC). All samples were analyzed at optimized chromatographic conditions and % RSD was calculated after calculating the average area of the three concentrations. ^16^

### Accuracy

Accuracy study is an assay method where percentage recovery is calculated. The study was performed by the addition of three different known concentrations at three levels 50%, 100% and 150%. Three injections of each concentration (1.5, 2 and 2.5 μg/ml) were injected and analyzed under the optimized chromatographic conditions. % RSD was calculated for all samples. ^17^

### Limit of Detection and Limit of Quantification

The Limit of Detection (LOD) and Limit of Quantification (LOQ) were determined from the linearity curve by measuring the ratio of signal to noise. LOD is the concentration that comes as a result of measuring the 3:1 signal-to-noise ratio. LOQ is the concentration that comes as a result of measuring the 10:1 signal-to-noise ratio. ^18^

### Robustness

The effect of intended changes that were made in chromatographic conditions like the composition of mobile phase, wavelength, column temperature and flow rate in the analytical method were studied. The changes considered for analysis are given below. ^19^

i. Organic phase ratio 33% and 37%
ii. Column temperature 22°C and 28°C
iii. Wavelengths 388 and 392 nm

### Bench-top stability studies

Benchtop stability was determined for analytical samples of the same concentration (3.5 μg/ml). The samples were kept at room temperature and injected three times at different time points (4, 8, 12 and 24 h). The peak area, retention time and %RSD were determined. ^20^

### Forced or stress degradation studies

Force degradation studies were performed to determine the stability of NTB in the developed analytical method by exposing it to various stress as discussed below. ^21^

***Acid degradation***

0.1 N HCl was added into 4 μg/ml of standard NTB solution, and the solution was subjected to heating by using the reflux condensing method for 60 min at 85°C temperature. One standard control sample was prepared in the same conditions by eliminating the heating step. Three replicates of each sample were injected by neutralizing with base.

***Base degradation***

0.1 N NaOH was added into 4 μg/ml of standard NTB solution, and the solution was subjected to heating by using the reflux condensing method for 60 min at 85°C temperature. One standard control sample was prepared in the same conditions by eliminating the heating step. Three replicates of each sample were injected by neutralizing them with acid.

***Oxidative degradation***

3% Hydrogen peroxide was added to 4 μg/ml NTB standard solution. Three replicates of each sample were injected.

***Thermal degradation***

2 μg/ml NTB standard solution was exposed to 105°C temperature for 24 h. Three replicates of each sample were injected. % assay and % RSD were calculated.

***Photolytic degradation***

1.5 μg/ml NTB standard solution was exposed to UV light for 24 h and the energy given was 200-watt hours/square meter. Three replicates of each sample were injected. The % assay and % RSD were calculated.

### Application of the validated analytical method

The developed analytical method was applied for assessing the encapsulation efficiency of optimized NLCs formulation. NLCs were prepared by melt emulsification sonication method. Formulation was centrifuged for 30 min at 22,000 rpm by maintaining the temperature at 4°C. After centrifugation, the pellet was collected and ruptured with methanol and necessary dilutions were made by using diluent. Placebo was prepared in a similar manner excluding drug and analyzed.

## Results and discussion

### Method optimization

**Figure 2.**
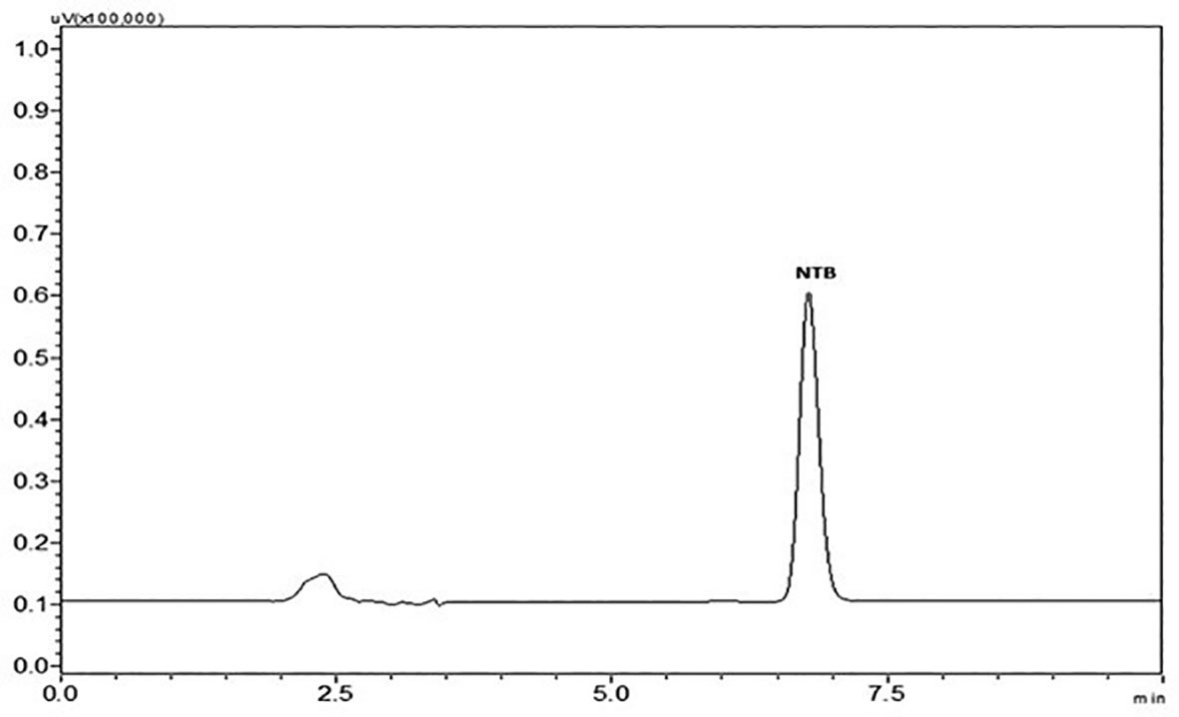
Chromatogram of Nintedanib (NTB) peak at optimized chromatographic conditions.

A stability indicating HPLC method for the determination of NTB in the formulation was developed and validated in the current investigation. The approach was focused primarily on lowering the amount of organic solvents used, making the mobile phase simple and feasible without the inclusion of buffer salts and shortening the run time. To determine whether the created approach of the analytical method was reliable for the analysis of NTB, it was validated as per ICH Q2(R1) recommended guidelines. The optimized method comprised of mobile phase ratio 35:65 (organic: aqueous) where the organic phase was ACN and the aqueous phase was 0.1% TEA and pH was adjusted to 3 with OPA and run time for 10 min. Chromatogram of the NTB peak with optimized chromatographic conditions is represented in Figure 2.

### Effect of mobile phase

Acetate buffer with pH 4.5 and phosphate buffer with pH 6.8 have not shown proper elution of the NTB. An increase in ACN in the mobile phase to 40 and 45% have shown less theoretical plate count which was not suggestable for a precise method. 0.1% TFA with more ACN ratio in the mobile phase has shown broader peaks and fronting was seen which is not acceptable. 0.1% TFA with less ACN ratio in the mobile phase has shown broader peaks with fronting and it was seen that the peak was eluted earlier with less retention time which is not acceptable. At lower ACN ratios, the NTB peak was found to be satisfactory. A sharp NTB peak was observed with 0.1% TEA with pH 3 adjusted with OPA, this might be due to the high polarity of NTB, and fronting was reduced due to the addition of 0.1% TEA. These findings led to the finalization of the procedure using 0.1% TEA and ACN as the mobile phase in a ratio of TEA:ACN (65:35%v/v). Chromatograms showing the effect of different mobile phases (A) 0.1% TEA:ACN (65:35%v/v), B) 0.1% TEA:ACN (55:45%v/v), C) 0.1% TEA:ACN (60:40%v/v), D) 0.1% TFA:ACN (60:40%v/v) and E) 0.1% TFA:ACN (55:45%v/v) are represented in Figure 16.

### Effect of column

To elute NTB, various columns like Luna, Phenomenex, Chromasol and Shimadzu C _18_ were tested. The increased affinity of NTB was seen in the chromasol C _18_ column as peaks obtained were larger and broad even at the high organic ratio in the mobile phase. NTB peaks obtained by Luna C _18_ column have shown less theoretical plate count. In Shimadzu C _18_ column NTB peaks have shown clear and sharp peaks without any noise and with high theoretical plate count. The sharp peaks in the Shimadzu C _18_ column may be due to the ability of high hydrophobic affinity towards Shimadzu column (as NTB is a hydrophobic drug) and core-shell technology ( www.Shimadzu.com). ^22^

### Effect of flow rate

When the flow rate was increased from 1 ml/min to 1.2 ml/min, the NTB peak shape not changed significantly. Hence, the flow rate was kept constant at 1 ml/min as it will be economical when compared to 1.2 ml/min.

### Effect of column oven temperature

The temperature of the column oven had no discernible effect on the shape of the peak when it was increased from 25°C to 40°C. Therefore, the temperature of the column oven was regulated at around 25°C (room temperature), as greater temperatures could shorten the column’s lifespan.

### Method validation

**Figure 3.**
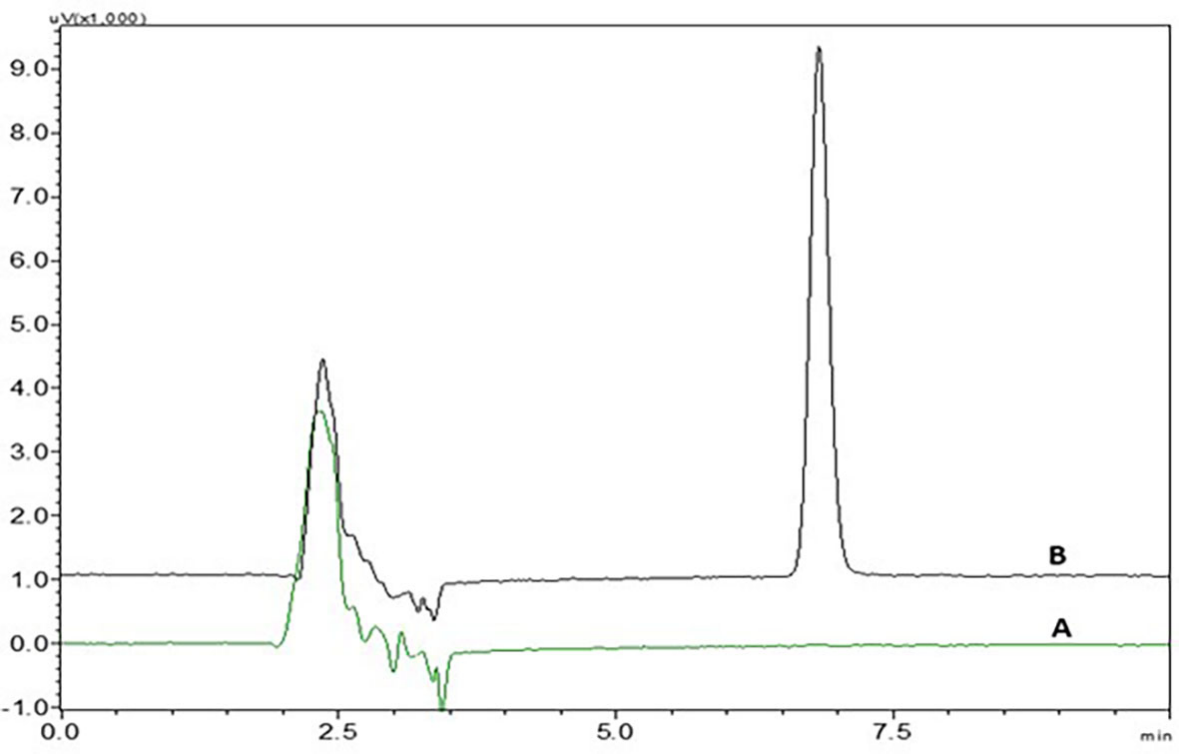
Chromatograms of A) Nintedanib (NTB) and B) Blank.

There was no interference from the peaks of the placebo, diluent and standard NTB solution at the retention time of NTB, indicating that the suggested analytical method was precise and specific in the quantification of NTB in the formulation ( Figure 3 and 15). The correlation coefficient showed that the developed analytical method was linear from the range of 0.5 μg/ml to 4.5 μg/ml. System suitability, precision and accuracy studies revealed that the technique produces precise findings for analyzing NTB. The % RSD of the formulation which was discovered to be less than 2% when compared with the optimized analytical method, provided further evidence of the analytical method’s robustness. Variations in flow rate, wavelength, column oven temperature, and mobile phase composition (% buffer) were made to test the method’s robustness, and their impact on the peak area, plate count, tailing factor, and retention duration was measured. All measured % RSD values were within the limit.

### System suitability

Parameters like theoretical plates, tailing factor, retention time and % RSD were calculated from peaks are tabulated in Table 2. All parameters were within the limits which shows that the developed analytical method was accurate and suitable for analyzing other samples.

**Table 2.** Results of validation parameters for analytical method (Mean ± SD).

	Method responses	Acceptance criteria		Observed	
System suitability	RSD of peak area (n=6)	RSD < 2.0 %		0.13	
	Tf _10%_	< 1.5		0.11	
	N	> 2000		6970.14	
	Retention time (min) (n=6)	RSD < 2.0 %		0.04	
Linear regression data	Linearity (μg/ml); (n=3)	-		0.5-4.5	
	Retention time (min)			6.77±0.00	
	Slope			y = 98912x + 50683	
	R ^2^			0.999	
Quantification				LOD	LOQ
				2.45	7.45
Precision	Sample	< 2.0 %		Interday	Intraday
	LQC			0.00	0.00
	MQC			0.40	0.40
	HQC			0.00	0.00
Accuracy	Initial conc. (μg/ml)	Observed mean conc. (μg/ml) n=3		Mean recovery (%)	
	1.5	1.49±0.04		99.39±0.46	
	2	2.01±0.03		99.38±0.25	
	2.5	2.47±0.03		97.92±0.15	

LQC: lower quality control, MQC: Middle quality control, HQC: Higher quality control, LOD: Limit of detection, LOQ: Limit of quantification.

### Specificity

There was no interference seen in the peaks of blank when compared to the sample solution and the chromatograms of blank and NTB showing without interference were represented in Figure 3.

### Linearity

**Figure 4.**
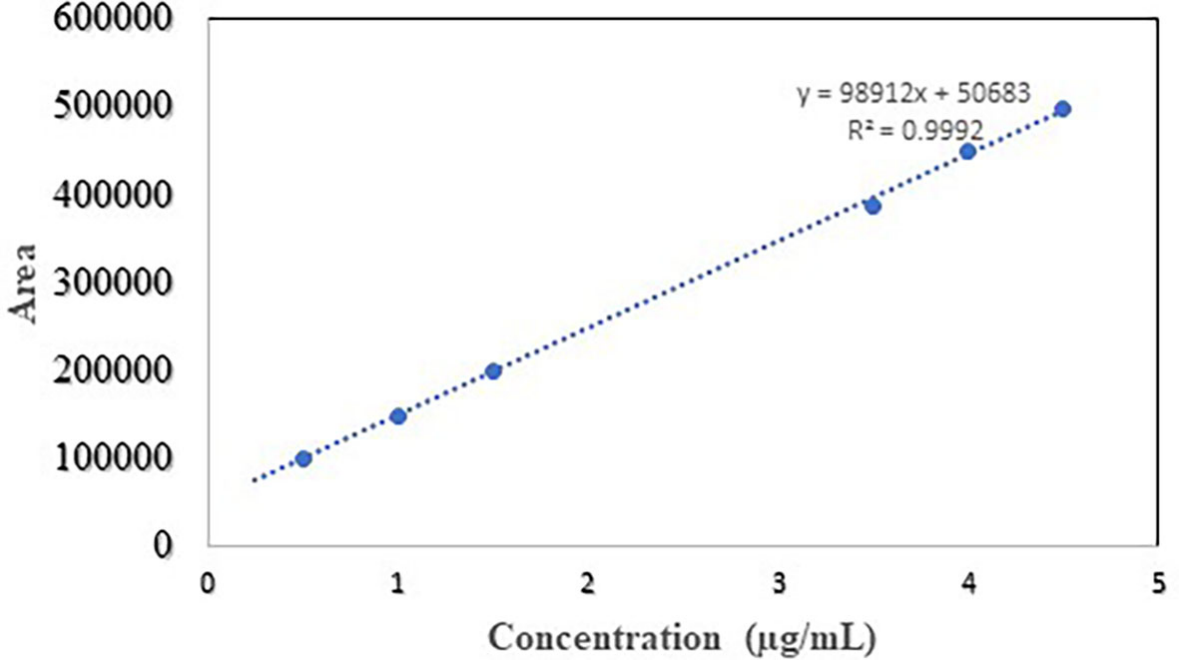
Linearity curve of Nintedanib.

The relation between the concentration of NTB samples and peak area to the developed method was directly proportional and proved the linearity. The linear equation obtained for analytical samples was given in Table 3 and the regression equation was y = 98912x+50683 whereas goodness of fit R ^2^ is 0.999. The calibration curve for analytical samples is given in Figure 4.

**Table 3.** Robustness and stability of the developed analytical method (Mean ± SD).

Robustness	Column temperature 22 (°C)	Area n=6	**Area**	**RSD**
			200242±897.41	0.00
	Column temperature 28 (°C)		200702±1011.78	0.00
	Detection wavelength 388(nm)		199223.5±766.55	0.00
	Detection wavelength 392 (nm)		197350.8±569.37	0.00
	Mobile phase 33 ACN		198489.33±196.92	0.00
	Mobile phase 37 ACN		197197.8±434.48	0.00
Benchtop stability	4 h	Area n=3	420628±879.27	0.20
	8 h		423293.7±1031.7	0.24
	12 h		410923.3±965.92	0.23
	24 h		438391.3±1355.60	0.30
Photolytic degradation	Exposed to UV lamp for 24 h		205711.7±222.03	0.10
Thermal degradation	105°C for 48 h		252078.7±549.65	0.21

ACN: Acetonitrile, UV: Ultra-violet lamp.

### Precision

Precision results have shown a close relationship between inter-day and intraday samples in all three LQC, MQC and HQC ranges. % RSD acceptance range for precision was less than 2% and all samples have fallen under this category and the results are given in Table 3.

### Limit of Detection and Limit of Quantification

The lowest concentration of the analyte in analytical samples which can be detected but not compulsorily quantitated is known as LOD. The lowest concentration of the analyte which can be quantified precisely is known as LOQ. The results of LOD and LOQ are given in Table 3.

### Robustness

It was shown that there was not much change in the results by changing the composition of mobile phase, wavelength, column temperature and flow rate. There was no distinct change in peak area and % RSD values of samples were in the acceptable range. %RSD and peak area values for analytical are given in Table 3.

### Bench-top stability studies

**Figure 5.**
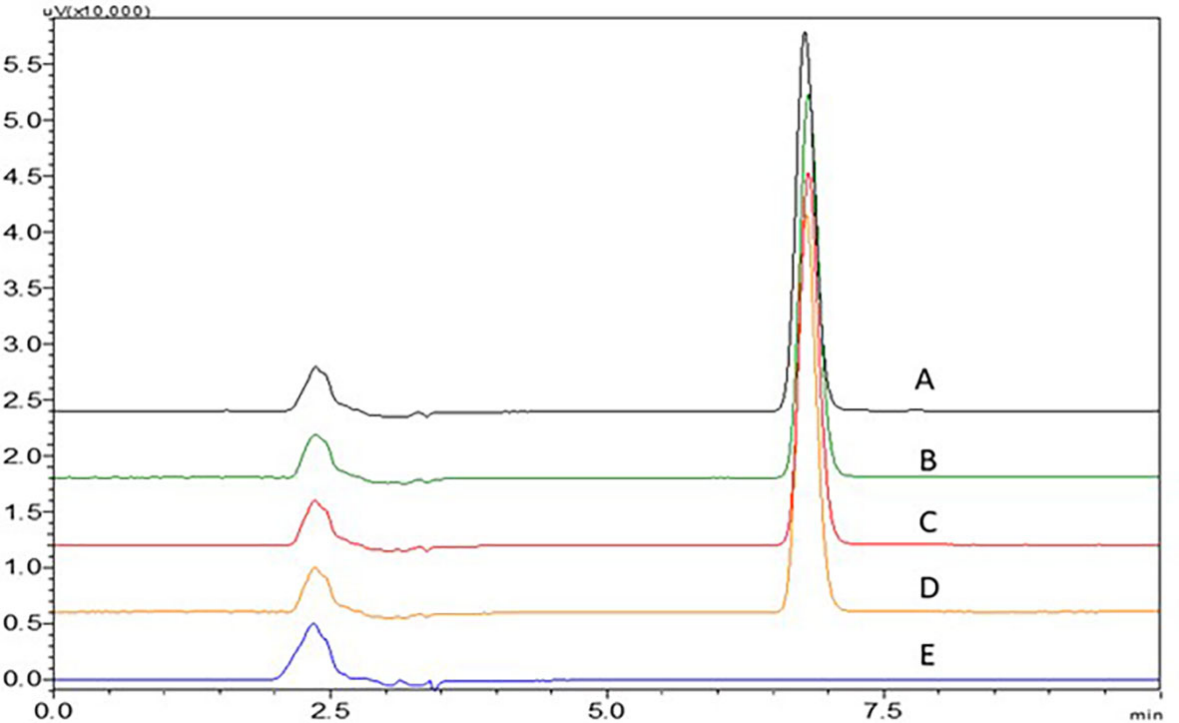
Chromatograms of Nintedanib (NTB) at A) 4 h, B) 8 h C) 12 h, D) 24 h showing stability of the NTB and E is blank chromatogram.

There was no change in the retention time of the peaks till 24 h, the chromatograms at different time intervals can be seen in Figure 5 and % RSD values were in an acceptable range which shows that NTB is stable, and the peak area, % RSD values are given in Table 3.

### Forced or stress degradation studies

Force degradation studies were carried out to determine if any degradants were formed due to exposure to stress conditions.

***Acid degradation***

**Figure 6.**
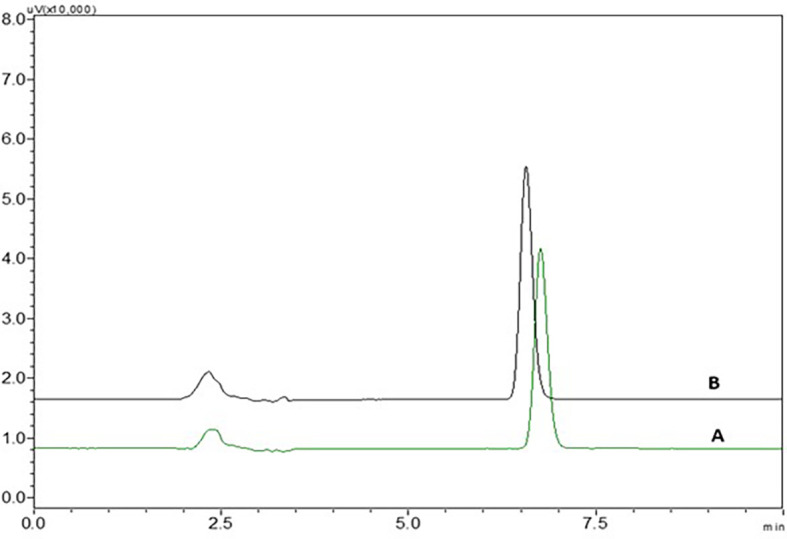
Chromatograms of A) Nintedanib (NTB) and B) NTB and acid degradants.

0.1N HCl treated NTB standard solutions which were kept at room temperature have not shown any degradant peaks. 0.1 N HCl treated NTB solutions which were subjected to 85°C temperature for 30 min have not shown any degradant peaks. There was a change in retention time (6.45±00) where the acid-treated sample was eluted slightly earlier than the NTB elution peak at optimized chromatographic conditions. % Assay of the acid degradation samples was found to be 86.62±2.48 and peaks are represented in Figure 6.

**Figure 7.**
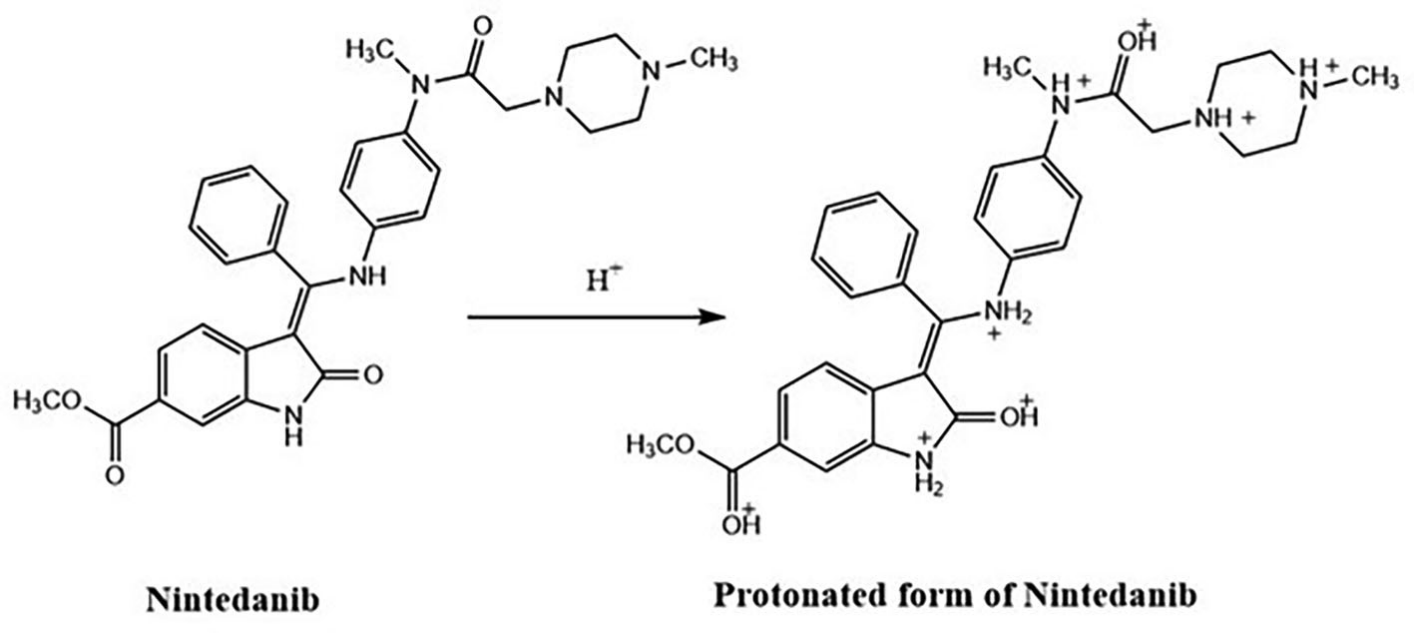
Degradant of Nintedanib (NTB) formed due to acid degradation.

Since there are five nitrogen atoms and three carbonyl groups there would have been protonation on the lone pair of electrons on the nitrogen and oxygen. Hence the resulted protonated structure has more or less similar but not the same Rt value compared to NTB standard peak. The degradant formed due to acid is given in Figure 7 and this acid degradation was supported by the literature. ^23^

***Base degradation***

**Figure 8.**
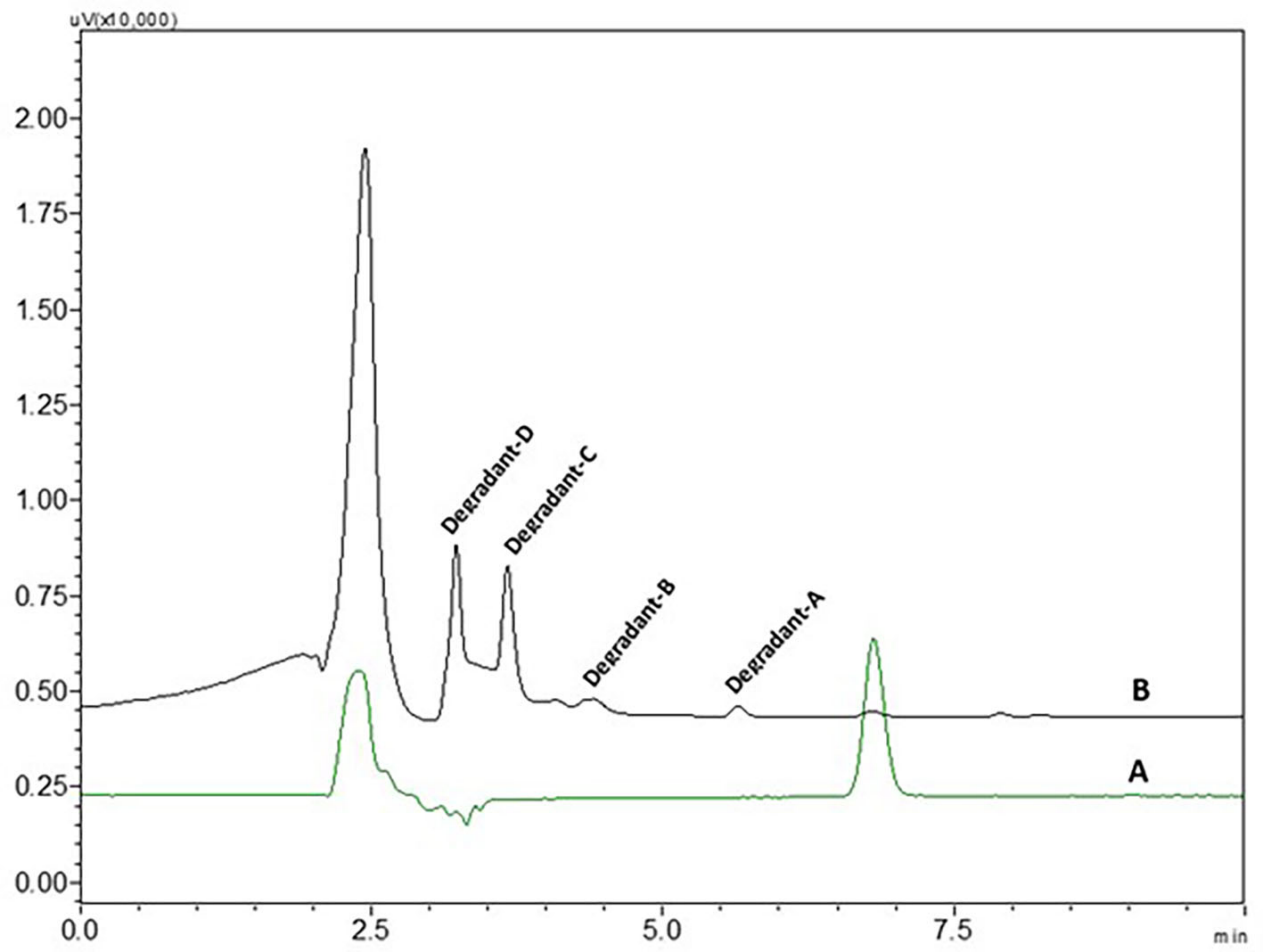
Chromatograms of A) Nintedanib (NTB) and B) NTB and base degradants.

0.1 N NaOH NTB standard solution treated NTB standard solutions which were kept at room temperature have not shown any degradant peaks, 0.1N NaOH treated NTB solutions that were subjected to 85°C temperature for 30 min have shown four degradant peaks and peaks are represented in Figure 8.

**Figure 9.**
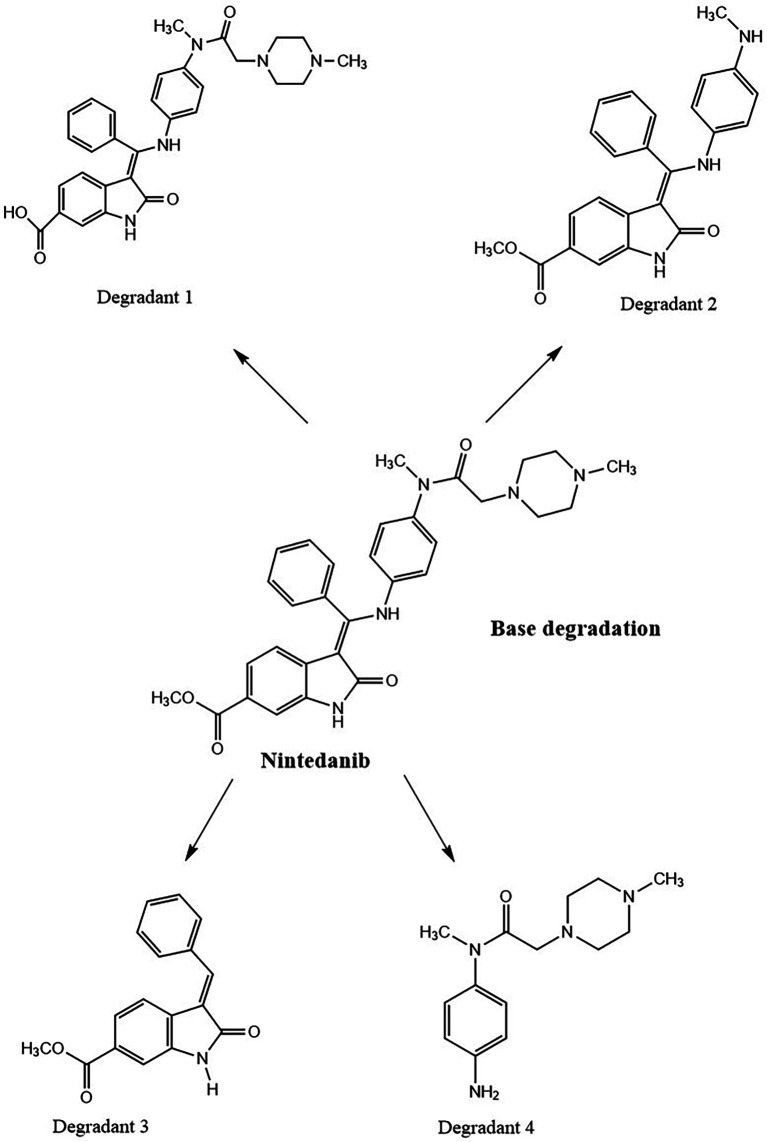
Degradants of Nintedanib (NTB) formed due to base degradation.

NTB is susceptible to undergo degradation in the presence of basic media. The carbonyl groups are attacked by hydroxyl ions via nucleophilic addition mechanism yielding the major degradants. Also, the alkene bond is susceptible to being cleaved. Degradants formed due to base are given in Figure 9. Degradation of NTB due to base/alkali was also reported in the literature. ^8^

***Oxidative degradation***

**Figure 10.**
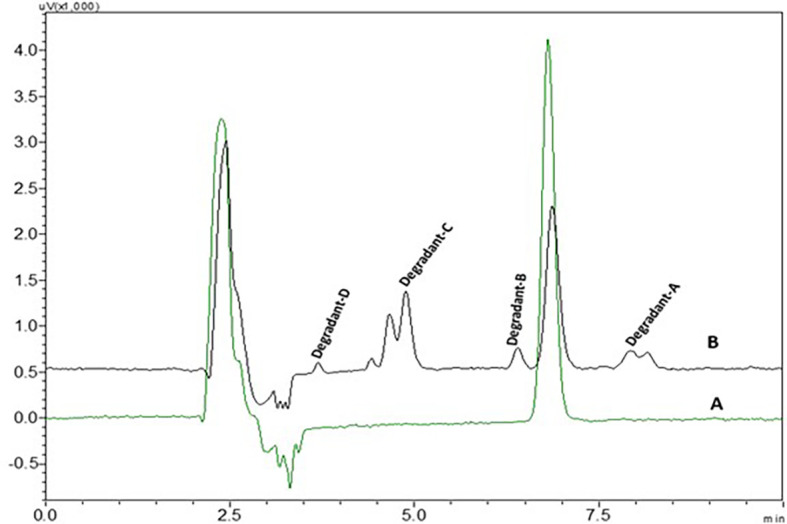
Chromatograms of A) Nintedanib (NTB) and B) NTB and peroxide degradants.

**Figure 11.**
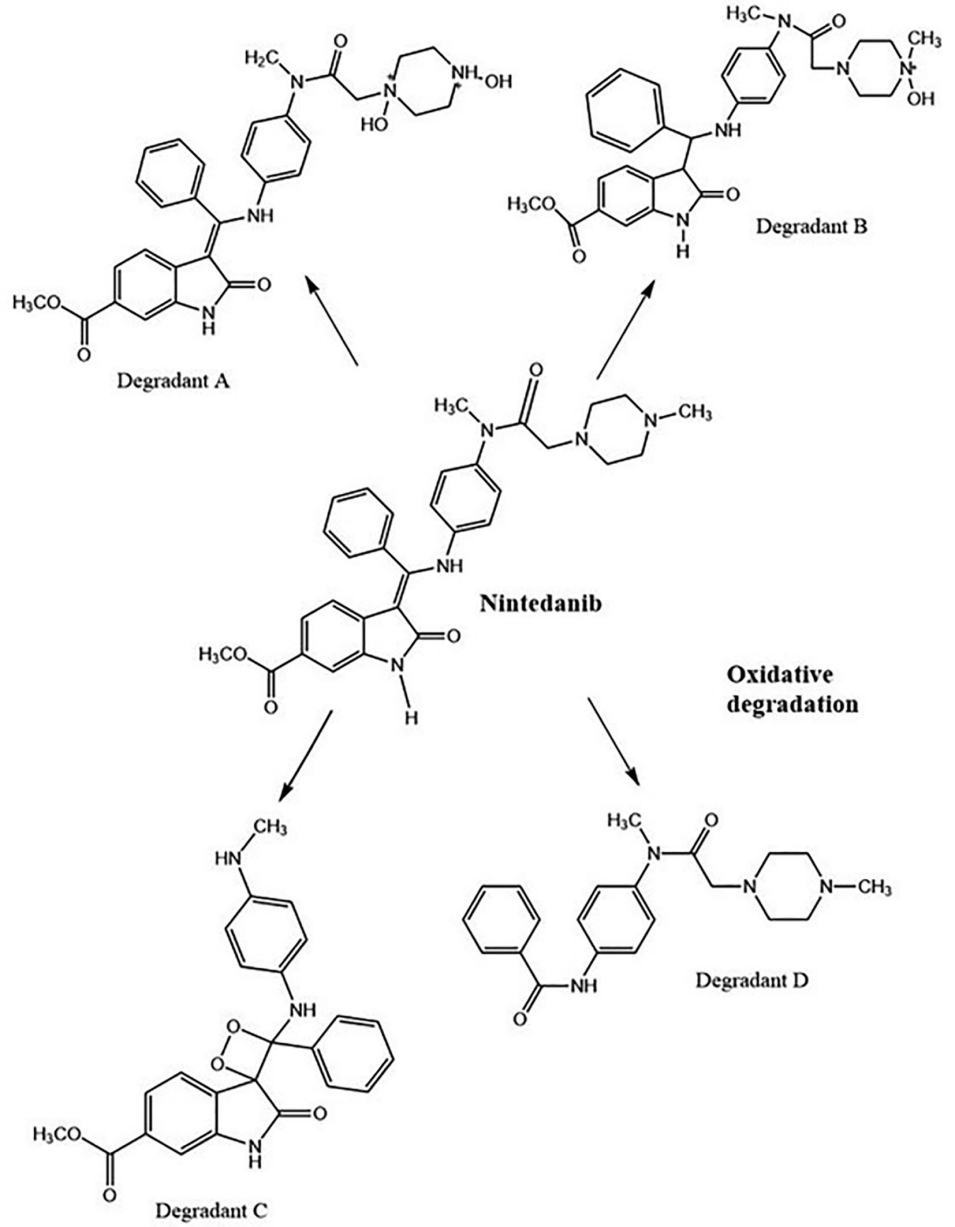
Degradants of Nintedanib (NTB) formed due to Oxidation.

3% hydrogen peroxide standard solution at ambient temperature for 24 h has shown four degradant peaks, % assay of the peroxide degradation samples was not calculated as the peak area values are very less than the linearity curve range. The degradant peaks are represented in Figure 10. The oxidative stressing of NTB is clearly showing the formation of four degradation products. The two nitrogen’s of piperazine undergoing N-oxidation and peroxidation of alkene is another major degradation product. Degradation of NTB due to oxidation is given in Figure 11 and oxidative degradation of NTB is also reported in the literature. ^9^

***Thermal degradation***

**Figure 12.**
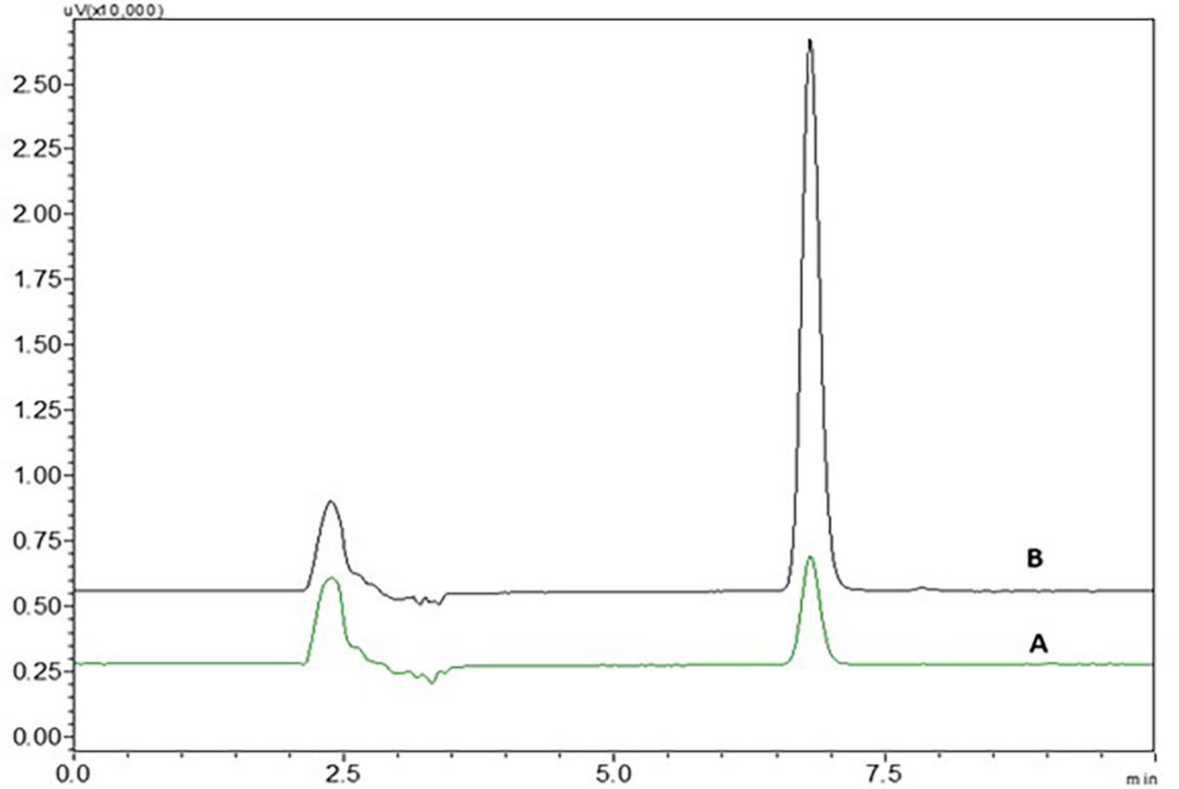
Chromatograms of A) Nintedanib (NTB) and B) NTB thermal degradation.

There were no degradant peaks observed in thermal degradation indicating thermal stability of NTB and the % assay of the thermal degradation samples was found to be 98.22±0.267. % RSD values and peak area were given in Table 3. And peaks are represented in Figure 12.

***Photolytic degradation***

**Figure 13.**
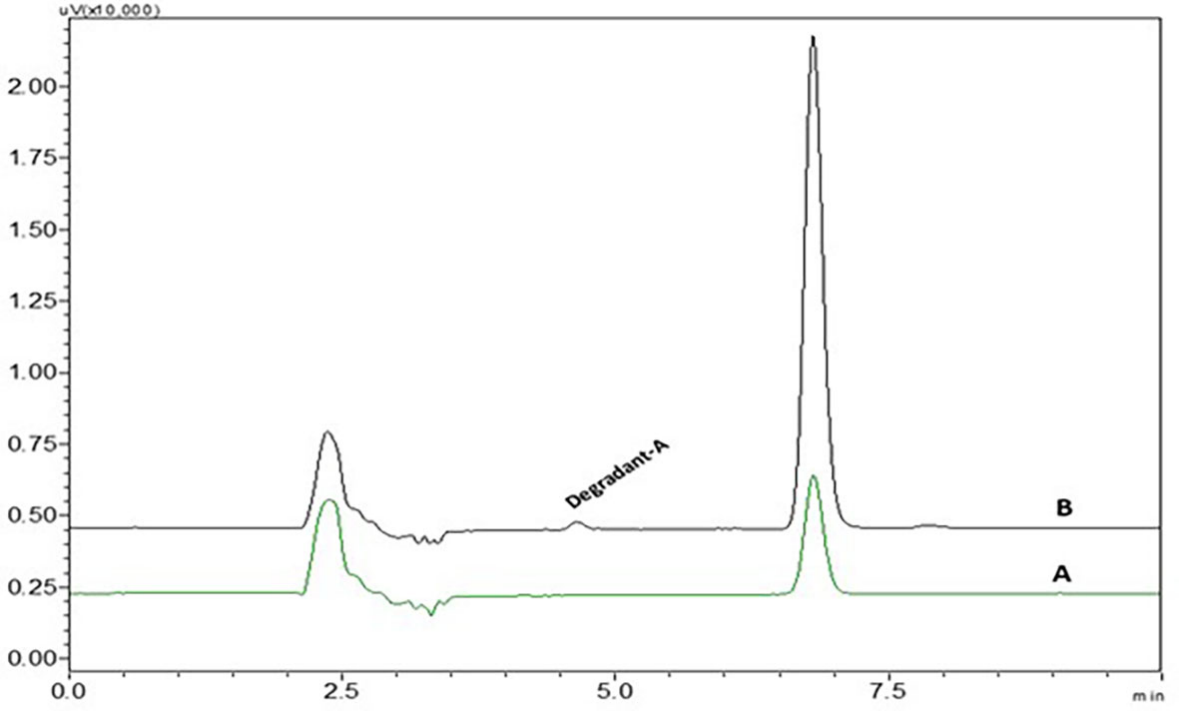
Chromatograms of A) Nintedanib (NTB) and B) NTB and photolytic degradant.

There was one very small degradant peak observed in photolytic degradation and the % assay of the photolytic degradation samples was found to be 95.7.3±0.137. % RSD values and peak area were given in Table 3. The degradant peak is represented in Figure 13. Most of the drug was stable to photolytic stress conditions but a small percentage of the drug has undergone degradation at the alkene bond.

**Figure 14.**
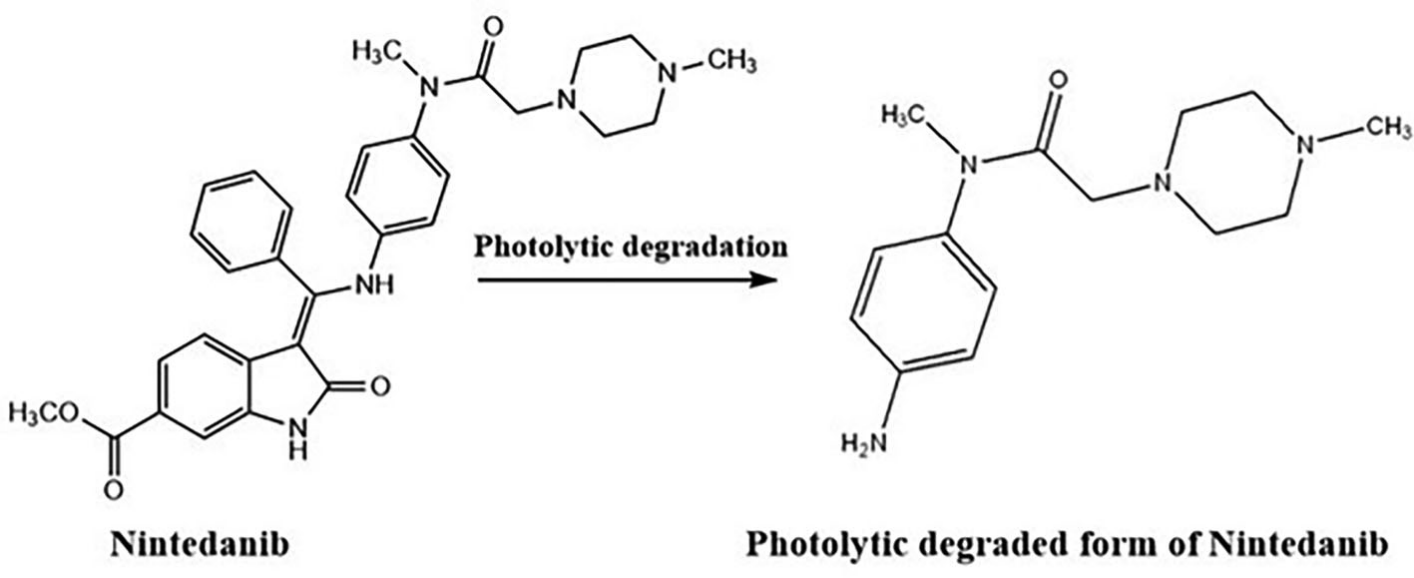
Degradant of Nintedanib (NTB) formed due to Photolytic degradation.

By injecting the stressed sample and unstressed sample into the HPLC instrument, the degradation peaks were identified. To ascertain the specificity of the NTB peak and peak purity of the corresponding substance, NTB was subjected to many stress conditions like thermal, light, acid, alkali, and oxidative stresses. To produce a measurable level of degradation, the stressing agent concentration and exposure time were optimized. The investigation was conducted to evaluate other degradant peaks as well as the purity of the NTB peak. It was discovered that NTB was more prone to alkali degradation, which was also reported in other literature. ^24^ The electrophilic attack of oxygen radicals which will promote the development of degradant products causes the oxidative degradation of NTB. UV rays may induce NTB to isomerize and result in the formation of degradant ( Figure 14). NTB was stable at room temperature which was confirmed by chromatograms which were taken at different time intervals till 24 h. The Stability-indicating RP-HPLC Method for NTB verified that the developed analytical method was specific for the determination or quantification of NTB and the retention time of NTB is free of interference from degradant peaks.

### Application of the validated analytical method

**Characterization of nanostructured lipid carriers**

The particle size of NLCs was found to be 290.5 ± 3.41 nm and the polydispersity index (PDI) was found to be 0.394 ± 0.028. From the PDI result it was confirmed that the formulation was monodisperse, whereas zeta potential was found to be -19.8±2.185 mV.

***Specificity of validated method***

**Figure 15.**
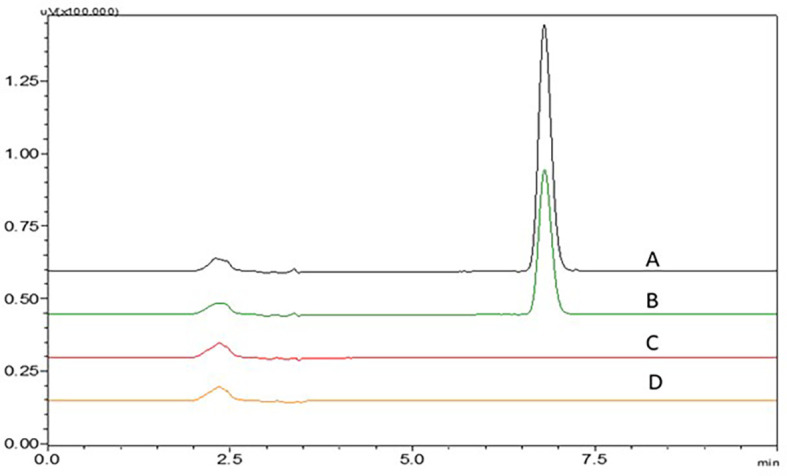
Chromatograms of A) Nintedanib (NTB) formulation, B) NTB standard, C) Placebo and D) Blank.

It was seen that the drug, the formulation’s excipients, and the blank peaks (methanol) did not interfere with one another. Chromatograms of formulation, placebo and blank are represented in Figure 15.

**Figure 16.**
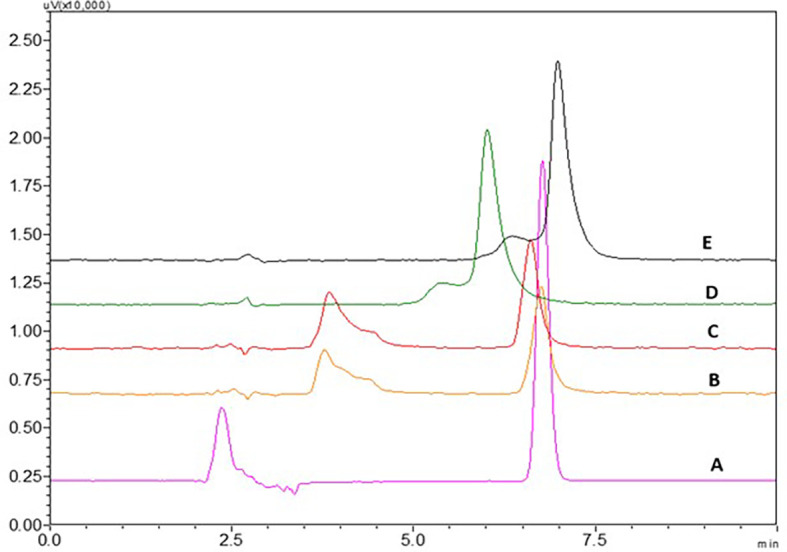
Chromatograms showing effect of different mobile phases A) 0.1% TEA:ACN (65:35% v/v), B) 0.1% TEA:ACN (55:45% v/v), C) 0.1% TEA:ACN (60:40% v/v), D) 0.1% TFA:ACN (60:40% v/v), E) 0.1% TFA:ACN (55:45% v/v).

***Encapsulation efficiency***

Encapsulation efficiency was found to be 88.241±0.155% and the % RSD value was in the acceptable range with the value of 0.176. There was no change in retention time and theoretical plate count in all the samples.

The amount of NTB in the NLCs formulation was measured using the RP-HPLC technique established in the current investigation. It was discovered that the developed analytical method was precise because there was no interference from a blank peak, placebo peak and NTB standard peak. It was difficult to recover the drug from the formulation excipients, ^25^ but the current developed analytical method demonstrated a recovery that was well within the allowable range.

The outcomes of the present analytical method point to the possibility of effectively analyzing the amount of NTB in the NLCs formulation.

## Conclusion

The developed HPLC method for determining NTB was found to be sensitive, precise, and repeatable. The technique was verified against the various concentration ranges of NTB (0.5-4.5 μg/ml. It was discovered to provide good accuracy and precision for the quantification of the NTB. The procedure was very reliable because the outcomes were unaffected by minor adjustments to the instrument’s settings or the composition of the mobile phase. The developed analytical method was discovered to have lower LOD and LOQ than the methods that were previously published. Also, it was discovered that the approach suggested in this study was very specific for the identification of NTB even when other formulation excipients were present. Our study will be helpful for the quantitative evaluation of NTB by this stability-indicating HPLC method because there are currently no publications on this topic with complete degradation studies which are advantageous for the quantification of NTB in bulk drugs and pharmaceutical preparations.

## Data Availability

Figshare: Validation of RP-HPLC for Nintedanib, https://doi.org/10.6084/m9.figshare.23565852.v1. ^26^ This project contains the following underlying data:
- HPLC_analytical validation_2023.xlsx Data are available under the terms of the Creative Commons Attribution 4.0 International license (CC-BY 4.0).
