# Supplementary material for: A stability indicating method development and validation of a rapid and sensitive RP-HPLC method for Nintedanib and its application in quantification of nanostructured lipid carriers
Source: F1000Res. 2024 Jun 10;12:1389. Originally published 2023 Oct 20. [Version 2] doi: 10.12688/f1000research.138786.2 (PMC11214665; doi:10.12688/f1000research.138786.2)
Supplement: Peer review history

# Reviewer report: Reviewer response for version 2

**DOI:** 10.5256/f1000research.167578.r289203

**Published:** 2024-06-29

**Authors:** Mohana Vamsi Nuli

**Affiliations:** 1 Raghavendra Institute of Pharmaceutical Education and Research, Anantapur, Andhra Pradesh, India

I am accepting the article

Is the work clearly and accurately presented and does it cite the current literature?

Yes

If applicable, is the statistical analysis and its interpretation appropriate?

Partly

Are all the source data underlying the results available to ensure full reproducibility?

Yes

Is the study design appropriate and is the work technically sound?

No

Are the conclusions drawn adequately supported by the results?

Yes

Are sufficient details of methods and analysis provided to allow replication by others?

Yes

Reviewer Expertise:

NA

I confirm that I have read this submission and believe that I have an appropriate level of expertise to confirm that it is of an acceptable scientific standard.

# Reviewer report: Reviewer response for version 1

**DOI:** 10.5256/f1000research.152014.r272267

**Published:** 2024-05-23

**Authors:** Mohana Vamsi Nuli

**Affiliations:** 1 Raghavendra Institute of Pharmaceutical Education and Research, Anantapur, Andhra Pradesh, India

# Page-7: Chromatograms of A and B are altered. 

 # Page-7: There is no placebo chromatogram 

 # Page-8: LOD and LOQ concentrations are in ppm? But standard concentration is 1ppm?

 # Page-9: Solution stability peak area is double than Robustness and other peak areas? Supposed to be the same peak area?

 # Page-16: Chromatogram B and C with increase in ACN when compared with, there is no change in NTB retention time. Also, Blank peak retention time increased with increase in ACN which supposed to decrease the Retention time.

Is the work clearly and accurately presented and does it cite the current literature?

Yes

If applicable, is the statistical analysis and its interpretation appropriate?

Partly

Are all the source data underlying the results available to ensure full reproducibility?

Yes

Is the study design appropriate and is the work technically sound?

No

Are the conclusions drawn adequately supported by the results?

Yes

Are sufficient details of methods and analysis provided to allow replication by others?

Yes

Reviewer Expertise:

NA

I confirm that I have read this submission and believe that I have an appropriate level of expertise to confirm that it is of an acceptable scientific standard, however I have significant reservations, as outlined above.

# Response

**Published:** 2024-06-05

**Authors:** Usha Yogendra Nayak

**Affiliations:** Department of Pharmaceutics, Manipal Academy of Higher Education, Manipal, Karnataka, India

**Reviewer Comment 1: **# Page-7: Chromatograms of A and B are altered.

**Answer to comment 1: **  In this chromatogram, peak A is blank methanol, and peak B is Nintedanib.

 Peak B chromatogram results from the Nintedanib in the sample, which causes variations in retention duration, intensity, and composition. The blank peak in the chromatogram is caused by methanol. By distinguishing it from impurities and background noise, this distinction guarantees precise identification and quantification of the analyte.

**Reviewer Comment 2:** # Page-7: There is no placebo chromatogram.

**Answer to the comment 2:**   As in figure 1 is only of method development the placebo peak is not given only pure Nintedanib drug chromatogram is given, the placebo peak is given in figure 15 where the drug from nano structured lipid carriers was determined.

**Reviewer Comment 3:** # Page-8: LOD and LOQ concentrations are in ppm? But standard concentration is 1ppm?

**Answer to comment 3: ** In the linearity curve, Nintedanib was found to have LOD of 2.45 μg/ml and LOQ of 7.45 μg/ml.

**Reviewer Comment 4:** # Page-9: Solution stability peak area is double than Robustness and other peak areas? Supposed to be the same peak area?

**Answer to comment 4:** Stability solutions chromatograms and robust chromatograms were done at different concentration ranges. Hence the areas are different.

**Reviewer Comment 5:**  # Page-16: Chromatogram B and C with increase in ACN when compared with, there is no change in NTB retention time. Also, Blank peak retention time increased with increase in ACN which supposed to decrease the Retention time.

**Answer to comment 5:** The consistent retention duration of NTB implies that variations in ACN content do not substantially impact it, possibly because of its unique chemical characteristics. On the other hand, the conventional trend of decreased retention with rising ACN concentration is overridden by an increase in the blank peak's retention time with greater ACN, which suggests distinct interactions between the impurities and the stationary phase or a change in solubility dynamics.

# Reviewer report: Reviewer response for version 1

**DOI:** 10.5256/f1000research.152014.r217521

**Published:** 2023-11-27

**Authors:** Rubi Mahato

**Affiliations:** 1 Wilkes University, Wilkes-Barre, Pennsylvania, USA

Reviewer’s Comments:

**Title: **A stability indicating method development and validation of a rapid and sensitive RP-HPLC method for Nintedanib and its application in quantification of nanostructured lipid carriers

 Overall, this research article is very well presented and provided with detailed research methodology and outcomes. Authors have provided elaborated procedures for each experiment done, and have provided the data and appropriate figures for all of them. I also like how the authors have listed the different types of columns they have tried before finalizing to use one. Also, the quantifiable range of Nintedanib (μg/ml) concentrations are provided.

 The stability studies of Nintedanib in presence of various factors such as acid, base, oxidation, elevated temperature, and light are provided as well. Moreover, authors have evaluated their newly developed RP-HPLC in evaluation of encapsulation efficiency of nanostructured lipid carrier, which serves as a validation of their newly developed method.

 Taken together, the findings are new, and a reliable and accurate method was developed to quantify the drug Nintedanib.

 Decision: accept as it is.

Is the work clearly and accurately presented and does it cite the current literature?

Yes

If applicable, is the statistical analysis and its interpretation appropriate?

Yes

Are all the source data underlying the results available to ensure full reproducibility?

Yes

Is the study design appropriate and is the work technically sound?

Yes

Are the conclusions drawn adequately supported by the results?

Yes

Are sufficient details of methods and analysis provided to allow replication by others?

Yes

Reviewer Expertise:

Synthesis of platform molecules, naturopathy, homeopathic treatments, curriculum research (Pharmaceutical Sciences )

I confirm that I have read this submission and believe that I have an appropriate level of expertise to confirm that it is of an acceptable scientific standard.
